# Vulnerability and resilience to prenatal stress exposure: behavioral and molecular characterization in adolescent rats

**DOI:** 10.1038/s41398-023-02653-6

**Published:** 2023-11-22

**Authors:** Kerstin Camile Creutzberg, Veronica Begni, Rodrigo Orso, Francisco Sindermann Lumertz, Luis Eduardo Wearick-Silva, Saulo Gantes Tractenberg, Moira Marizzoni, Annamaria Cattaneo, Rodrigo Grassi-Oliveira, Marco Andrea Riva

**Affiliations:** 1 Department of Pharmacological and Biomolecular Sciences, University of Milan, Milan, Italy; 2 School of Medicine, Pontifical Catholic University of Rio Grande do Sul, Porto Alegre, RS Brazil; 3 Biological Psychiatry Unit, IRCCS Istituto Centro San Giovanni di Dio Fatebenefratelli, Brescia, Italy; 4 Lab of Neuroimaging and Alzheimer’s Epidemiology, IRCCS Istituto Centro San Giovanni di Dio Fatebenefratelli, Via Pilastroni, 4, Brescia, 25125 Italy; 5 Translational Neuropsychiatry Unit, Department of Clinical Medicine, Aarhus University, Aarhus, Denmark

**Keywords:** Molecular neuroscience, Psychology

## Abstract

Exposure to stress can lead to long lasting behavioral and neurobiological consequences, which may enhance the susceptibility for the onset of mental disorders. However, there are significant individual differences in the outcome of stress exposure since only a percentage of exposed individuals may show pathological consequences, whereas others appear to be resilient. In this study, we aimed to characterize the effects of prenatal stress (PNS) exposure in rats at adolescence and to identify subgroup of animals with a differential response to the gestational manipulation. PNS adolescent offspring (regardless of sex) showed impaired emotionality in different pathological domains, such as anhedonia, anxiety, and sociability. However, using cluster analysis of the behavioral data we could identify 70% of PNS-exposed animals as vulnerable (PNS-vul), whereas the remaining 30% were considered resilient (PNS-res). At the molecular level, we found that PNS-res males show a reduced basal activation of the ventral hippocampus whereas other regions, such as amygdala and dorsal hippocampus, show significant PNS-induced changes regardless from vulnerability or resilience. Taken together, our results provide evidence of the variability in the behavioral and neurobiological effects of PNS-exposed offspring at adolescence. While these data may advance our understanding of the association between exposure to stress during gestation and the risk for psychopathology, the investigation of the mechanisms associated to stress vulnerability or resilience may be instrumental to develop novel strategies for therapeutic intervention.

## Introduction

Stress is an important determinant of human behavior and physiology, which may cause health problems in the long-term [1]. Exposure to stress frequently leads to behavioral and molecular alterations, which can be harmful depending on the timing, duration, and the intensity of the stressful situation [2–5]. Within this context, exposure to stress during gestation is known to increase the risk for different pathologic conditions, including mental disorders. Indeed, a healthy environment during the prenatal period is crucial for the developing organism that is highly susceptible to internal and external stimuli [6]. Exposure to stress during pregnancy may alter fetal development and produces molecular and functional changes in different brain structures leading to behavioral abnormalities in the offspring [7, 8].

We have previously shown that exposure to prenatal stress (PNS) produces increased anxiety-like behavior and cognitive impairments, which are associated with reduced expression of brain derived neurotrophic factor, alterations of the HPA axis and stress responsiveness, enhanced inflammation as well as long-term epigenetic changes [8–14].

However, there are important differences in the outcome of stress exposure. First, while some individuals exposed to adverse experiences are susceptible and develop a pathological condition, others appear to adapt and be resilient [15]. The manifestation of one or the other will depend on the genetic background, the environment, the interaction of these two factors, as well as the individual’s sex [16]. Moreover, the exposure to stress may affect distinct psychopathological domains, as emotional, cognitive, and social, which are shared among different mental disorders. Interestingly, the onset of stress-induced conditions, peaks between mid and late adolescence and become fully manifest during the transition from adolescence to adulthood [17–19]. Accordingly, some of the long-term alterations produced by PNS exposure peak around adolescence or in early adulthood [7, 9–11, 13].

Here we identified animals with distinct clusters of behavioral symptoms emerging in adolescence after exposure to prenatal stress and we characterized some neurobiological alterations associated with vulnerability or resilience to the stress exposure during gestation.

## Methods

### Experimental design and animal housing

Adult nulliparous male and female Wistar rats (*Rattus norvegicus*) were purchased from the Center for Experimental Biological Models (CeMBE) at the Pontifical Catholic University of Rio Grande do Sul, Brazil, and were left undisturbed in the animal facility for 10 days before the beginning of the experiment. Animals were kept in an environment with controlled temperature (21 ± 1 °C) and humidity (55 ± 5%) under a 12 h/12 h light/dark cycle (lights on at 6 am) with food and water *ad libitum* during the whole experiment. Cages were weekly changed by the animal facility staff. After acclimatization, animals were breaded (1 male and 2 females) for 48 h. Females were left together until the gestational day (GD) 14. At GD14 dams were single-housed and randomly allocated to the control (CT) or to the prenatal stress (PNS) group. PNS dams were exposed to a restraint stress protocol from GD14 to delivery while CT dams were left undisturbed. At postnatal day (PND) 0, the day of birth, litters were culled to 8 pups (4 males and 4 females when possible). Pups were then left undisturbed to prevent unnecessary manipulations until PND21 when they were weaned and housed in groups of 2/3 per cage. A total of 76 offspring animals were used in this study (16 CT males, 14 CT females, 30 PNS males, 18 PNS females), and were generated in two separate cohorts of animals. Behavioral assessment was carried out during adolescence (PND35-39). The stress model used here has been previously used in several studies of the research group (see introduction). All procedures included in this study were conducted in accordance with the Guide for the Care and Use of Laboratory Animals from the National Institute of Health (NIH) and were approved by the Ethics Committee on the Use of Animals of the Pontifical Catholic University of Rio Grande do Sul, under the protocol code #8922.

### Prenatal stress protocol

Dams from the PNS group were exposed to a restraint stress protocol during the last week of pregnancy (GD14 to delivery) as previously published [12]. The protocol was performed 3 times a day (starting at 9 am, 12 pm, and 5 pm ± 2 h) under bright light (1.500 lux), and each session lasted 45 min. Dams were placed into transparent Plexiglas cylinders (20 cm length × 9 cm diameter × 9 cm height) with adjustable closures that could be regulated depending on the animal’s size. Dams were weighed in intercalated days to access any stress effects on gain weight during pregnancy.

### Behavioral assessment

Maternal behavior was assessed in intercalated days between PND1 and PND9 and results are presented as the average of all days. Regarding the offspring, social interaction (SI), sucrose preference (SP), and novelty suppressed feeding (NSF) tests were performed between PND35 to PND39. All tests were video-recorded, and each video was analyzed by two researchers that were blind to the animal’s condition. An average of both researchers was used as the final scores. All tests were performed during the light phase. The apparatus was always cleaned with 70% ethanol between animals. A detailed description of behavioral evaluation can be found in the Supplementary Material.

### RNA extraction and transcriptional analysis

All animals were sacrificed by decapitation 2 days after the end of behavioral testing. The brain was quickly removed and free-hand-dissected for the collection of the prefrontal cortex (PFC), ventral (VH) and dorsal (DH) hippocampus, and amygdala (Amy). Dissected tissues were snap-frozen in dry ice and stored at −80 °C until molecular analysis. Total RNA was extracted from all brain regions (PFC, VH, DH, and Amy) from all animals. RNA extraction was performed using the RNeasy Mini Kit (#74104 Qiagen) according to the manufacturer’s protocol. RNA concentration was measured at NanoDrop spectrophotometer (Thermo Fisher) and further diluted at 10 ng/ul for quantitative real-time polymerase chain reaction (qRT-PCR) (CFX384 real-time system, Bio-Rad Laboratories). All samples were run in a 384 wells plate in triplicates with both ß-Actin and GAPDH as internal controls (housekeeping gene). Primers and probes were purchased from Thermo Fisher Scientific or Eurofins Genomics, and their ID’s or sequences are shown in Table S1. The efficiency corrected model was used for qRT-PCR analysis, in which the amplification efficiencies of target and housekeeping genes were considered [20]. Data are presented as fold change % compared to the CT group (set at 100%).

### Statistical analysis

Data were analyzed using IBM SPSS Statistics v.27 and GraphPad Prism 9. Student’s t-test and two-way ANOVA (for behavior direction) were used to perform statistical analyses related to the dams (CT x PNS dams). In the offspring, differences between groups were analyzed using a two-way ANOVA with sex (male and female) and stress (CT and PNS) factors. In the case of an interaction between sex and stress, Tukey’s *post hoc* test was used. To investigate the possible alterations between vulnerable and resilient animals, one-way ANOVA was performed. Data are presented as group mean ± standard error of the mean (SEM). The graphs represent individuals as dots, and *p*-value < 0.05 was considered statistically significant. All experimental groups have shown similar variance for all behavior and molecular parameters. The sample size used in this work was based on previous experiments from the research group and took in account that the aim of this work was to identify animals that were vulnerable or resilient to prenatal stress. The sample size was in accordance with those estimated by power analysis using G∗power software [12].

Vulnerable and resilient animals were identified and classified with a two-step cluster analysis, which took into account the latency to eat the food in the NSF, the social preference in the SI, and the sucrose preference in the SP test. The cluster analysis was performed without predefining a number of clusters, using the Schwarz information criterion (BIC) and the log-likelihood method as a distance measure [21].

A *Z*-score was calculated considering the immediate early genes (IEGs) that were analyzed with qRT-PCR (*Arc*, *Npas4*, *Cfos*, and *Zif-268*) to have an integrated overview of the molecular analysis. The individual *z*-score per animal in each gene was obtained by subtracting the group average value from the sample value and then dividing it by the group’s SD. Next, the Z-Activation scores were obtained by averaging the *z*-scores of all IEGS. The Z-Activation was calculated only when the qRT-PCR results from at least three genes were available.

To correlate any phenotypical output with gene expression changes we calculated Pearson’s coefficients, and a linear regression *t* test was used to check whether the correlation coefficient was significantly different from 0. Finally, the integrated analysis of brain activation, was performed using R as previously published [22].

## Results

### Prenatal stress exposure leads to fragmented maternal care

**Fig. 1:**
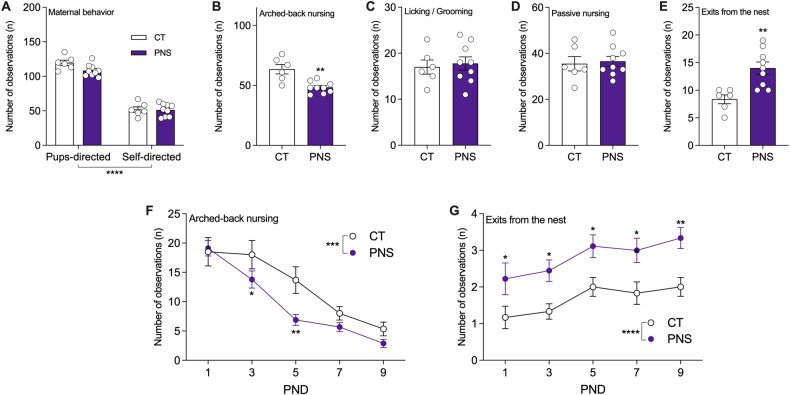
Analysis of maternal behavior after prenatal stress exposure. Maternal behavior was assessed every other day from PND1 to PND9 in control (CT) and stressed dams (PNS). The following parameters were measured: maternal behaviors directed to the pups or self-directed (**A**), arched-back nursing (**B**), licking and grooming (**C**), passive nursing (**D**), and nest exits (**E**). The total number of observations are shown in each panel. As for arched-back nursing and nest exits data are also shown across the observation period (panels **F** and **G**, respectively). The data are presented as the mean ± SEM of an average of observations from all days (panels **A**, **B**, **C**, and **D**) or from each day (panels **F** and **G**), *n* = 6–9 per group. Statistical analysis for panel (**A**): Effect of behavior direction *p* < 0.0001 (two-way ANOVA), independently of gestational exposure. Statistical analysis for panels (**B**–**D**): **p < 0.01, Student’s *t*-test. Statistical analysis for panels (**F**) and (**G**): area under the curve (AUC) for CT x PNS, ***p < 0.001, ****p < 0.0001; multiple *t* tests, *p < 0.05, **p < 0.01.

We first investigated if and how exposure to PNS may alter maternal behavior toward the newborn. As an overall view of maternal behavior, both CT and PNS dams showed an increase in pup-directed behaviors when compared to self-directed behaviors (F (1, 26) = 335.2, *p* < 0.0001; Fig. 1A). However, when looking at specific behaviors, such as the arched-back nursing, stressed dams showed a significant decrease of this important nursing posture, as compared to CT dams (t (13) = 4.077, *p* = 0.0013; Fig. 1B). No significant differences were found in other pup-directed behaviors, namely licking and grooming (Fig. 1C), and passive nursing (Fig. 1D). On the other end, PNS dams showed a fragmented maternal behavior by increasing the frequency of exits from the nest during the observation period (t (13) = 3.427, *p* = 0.0045; Fig. 1E).

Since PNS dams showed reduced arched-back nursing and increased nest exits, we performed a secondary analysis to evaluate those changes across the observation period. Dams exposed to PNS showed decreased overall arched-back nursing, as compared to CT dams based on the area under the curve (AUC) (t (13) = 4.800, *p* = 0.0003; Fig. 1F). Moreover, multiple *t* tests analysis revealed that this decrease was significant on postnatal days 3 and 5 (*p* = 0.046 and *p* = 0.0018, respectively). Regarding the nest exits, PNS dams showed an overall increase in the AUC (t (13) = 7.060, *p* < 0.0001; Fig. 1G). Multiple *t* tests analysis showed that PNS dams had increased exits from the nest on postnatal days 1, 3, 5, 7, and 9 (*p* = 0.028, *p* = 0.021, *p* = 0.021, *p* = 0.015, and *p* = 0.006, respectively). The PNS-induced changes on maternal behavior can persist up to PND15 (data not shown).

### Prenatal stress exposure induced emotional dysfunctions in adolescent offspring

**Fig. 2:**
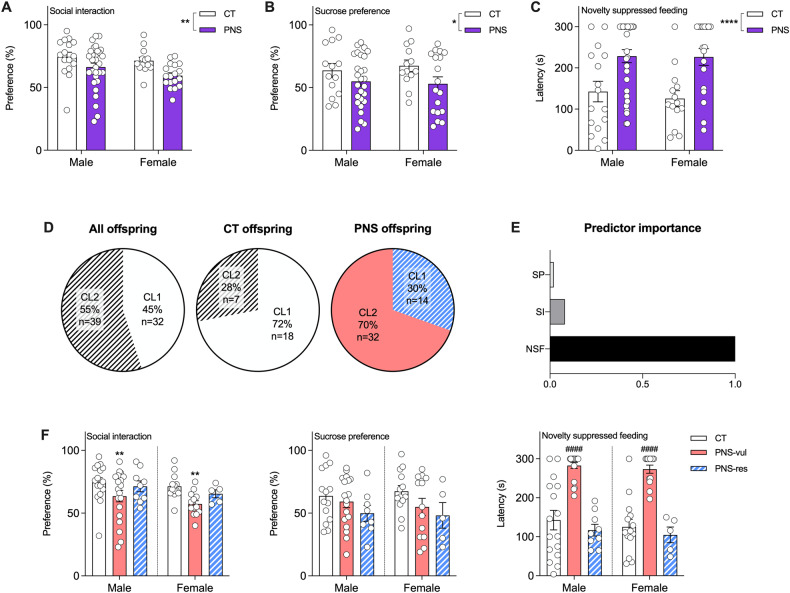
Behavioral phenotype of adolescent offspring exposed to prenatal stress and stratification into vulnerable and resilient subgroups. Pregnant dams were exposed to prenatal stress (PNS) or left undisturbed (CT). At adolescence (from PND35 to PND39) the resulting offspring were subjected to the following behavioral test: the social interaction test, displayed as percentage of social preference (panel **A**), the sucrose preference test, displayed as percentage of sucrose preference (panel **B**), and the novelty suppressed feeding test, displayed as latency time to eat the food (panel **C**). A two-step cluster analysis including the three behavioral tests (the latency to eat the food in the NSF, the social preference in the SI, and the sucrose preference in the SP test) from control (CT *n* = 25) and prenatally stressed (PNS *n* = 46) offspring was performed to identify subgroups of animals with different behavioral outcomes. The pie charts show the cluster distribution (with the percentages and the number of animals in each cluster) for all offspring combined, and for CT and PNS groups separately (panel **D**). Panel (**E**) displays the predictor of importance for cluster separation, with NSF having the highest predictor importance. Panel (**F**) shows the behavioral readouts for CT and PNS vulnerable (PNS-vul) and resilient (PNS-res) offspring of both sexes. Statistical analysis for panels **A**, **B**, and **C**: PNS effect, *p < 0.05, **p < 0.01, ****p < 0.0001, two-way ANOVA; *n* = 14 to 30 animals per group. Statistical analysis for panel (**F**): **p < 0.01 vs. the respective CT group and ^####^*p* < 0.0001, statistically different from the respective CT and PNS-res groups (one-way ANOVA, Tukey’s *post hoc)*; *n* = 5 to 19 animals per group. The data are presented as the mean ± SEM.

Stress exposure induced an overall emotional impairment, as seen by a decreased sociability, reduced sucrose preference, as well as increased latency to eat the food in both male and female rats tested during adolescence. In detail, we observed a significant main effect of stress exposure on sociability, as shown by the decreased social preference (F (1,72) = 12.25, *p* = 0.0048; Fig. 2A), on anhedonic behavior, demonstrated in the sucrose preference test (F (1,68) = 5.085, *p* = 0.0274; Fig. 2B), as well as in anxiety-like behavior, as evaluated in the novelty suppressed feeding test (F (1,74) = 20.68, *p* < 0.0001; Fig. 2C).

### Stratification of animals exposed to prenatal stress into vulnerable and resilient

Since individual animals may respond differently to stress exposure, we applied a two-step cluster analysis to identify possible heterogeneous behavioral responses to the gestational stress. The two-step cluster analysis identified two main clusters (CL1 and CL2) with good cluster separation (silhouette measure of cohesion and separation > 0.5). Among PNS animals, we found that 30% (14 out of 46) were identified as belonging to CL1, whereas the remaining 69% (32 out of 46) were classified into CL2 (Fig. 2D). Regarding the CT group, most of the animals felt within the CL1 (72%, 18 animals), and the remaining ones were considered to be part of CL2 (28%, 7 animals, Fig. 2D). As shown in Fig. 2E, the latency to eat in the NSF test had the highest predictor importance, followed by the social interaction preference obtained from the social interaction test, and sucrose preference, the latter having only minimal predictor importance for cluster separation.

Animals classified into CL1 showed lower levels of anxiety-like behavior in the NSF test and higher social interaction ratio than animals classified into CL2. The sucrose preference was similar in the offspring classified to both, CL1 and CL2. Therefore, we defined the animals within CL1 as resilient to PNS exposure (PNS-res), whereas we referred to animals within CL2 as vulnerable to gestational stress (PNS-vul).

The subsequent comparison between CT, PNS-vul, and PNS-res subgroups confirmed that only PNS-vul rats displayed significant dysfunctions in anxiety-related and social behaviors (Fig. 2F). Indeed, we observed a significant effect in the SI test (F (5, 69) = 2.656, *p* = 0.0228) with a significant reduction of social preference in PNS-vul rats, when compared to CT (CT vs PNS-vul, *p* = 0.0038 for males and *p* = 0.0012 for females), whereas PNS-res animals scored largely similar to the CT group (*p* = 0.99 for males and *p* = 0.96 for females). Similarly, we found a significant effect of gestational stress on anxiety-like behavior (F (5, 70) = 21.95, *p* < 0.0001), since male and female PNS-vul showed higher latency to eat during the NSF test, as compared to CT animals (CT vs PNS-vul, *p* < 0.0001 for both sexes). Moreover, we also found a difference between PNS-vul and PNS-res animals (PNS-vul vs PNS-res, *p* < 0.0001 for both sexes). No significant differences between CT, PNS-vul and PNS-res were instead found in the sucrose preference test.

### Analysis of activity-regulated genes in PNS vulnerable and resilient animals

Stress exposure is known to alter the activity state of different brain regions, which may modulate the function of specific brain circuits involved in emotional behavior [23]. To this purpose, we have selected four activity-dependent genes (also defined as immediate early genes - IEGs) that may be modulated by different synaptic inputs and that are also prototypical of two functional classes: regulatory transcription factors and “effector” proteins, which may directly modulate specific cellular functions [24]. The expression of these IEGs may represent a proxy for the activity state of brain structures that contribute to emotional liability, including the prefrontal cortex (PFC), the amygdala (AMY) as well as the ventral (VH) and dorsal (DH) hippocampus. The analysis for the single genes can be found in Figs. S1 (PFC), S2 (AMY), S3 (DH), and S4 (VH) and the detailed description of the changes for each gene is also reported in the Supplementary Material.

**Fig. 3:**
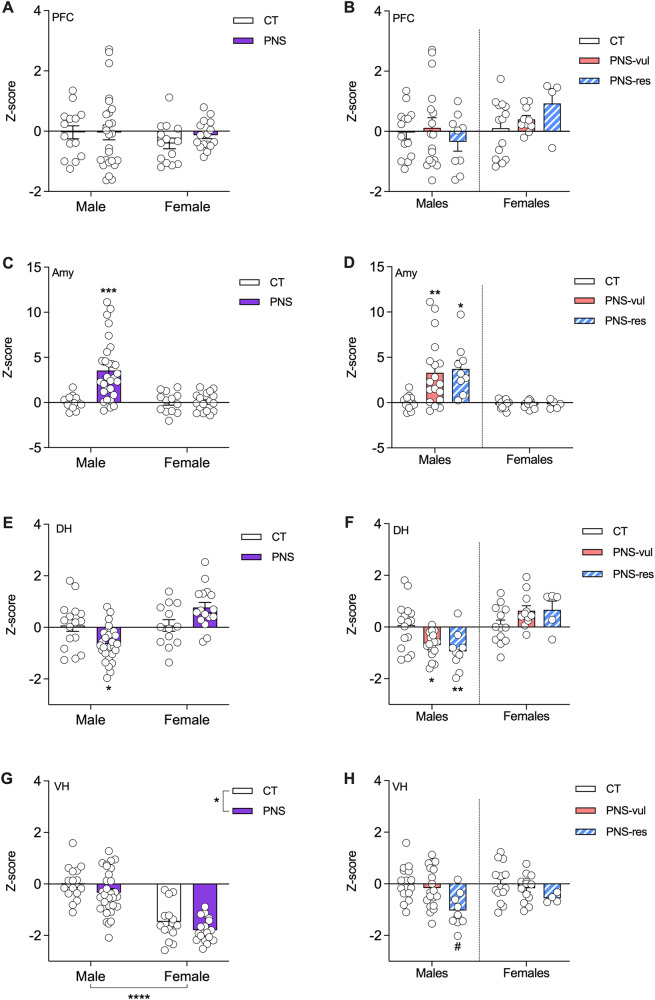
Analysis of Z-activation score in different brain regions following PNS exposure in male and female adolescent offspring. The Z-Activation score was calculated based on the analysis of IEGs expression (Arc, Npas4, Zif-268 and C-fos) in prefrontal cortex (**A**, **B**), amygdala (**C**, **D**), dorsal hippocampus (**E**, **F**), and ventral hippocampus (**G**, **H**) of male and female offspring. The analyses were performed as whole PNS group (**A**, **C**, **E**, and **G**) or after the separation in vulnerable (PNS-vul) and resilient (PNS-res) to the gestational manipulation (**B**, **D**, **F**, and **H**). Statistical analysis for panels **A**, **C**, **E**, and **G**: two-way ANOVA, sex effect, ****p < 0.0001; PNS effect, *p < 0.05; Tukey’s post hoc, *p < 0.05, ***p < 0.001, statistically different from CT male (*n* = 14 to 30 per group). Statistical analysis for panels **B**, **D**, **F**, and **H**: one-way ANOVA, Tukey’s post hoc, *p < 0.05, **p < 0.01, statistically different from CT male, ^#^*p* < 0.05, statistically different from the CT and PNS-vul male groups (*n* = 5 to 19 per group).

As shown in Fig. 3, we calculated a Z-activation score that took into consideration the expression of all four abovementioned genes as a global indication for the specific structure. When considering the prefrontal cortex, exposure to PNS did not produce any significant difference in males and females (Fig. 3A), even when considering the sub-clusters PNS-vul and PNS-res (Fig. 3B). Conversely, the analysis of the amygdala shows a significant sex x stress interaction (F (1, 68) = 9.298, *p* = 0.0033). Post hoc analysis showed that PNS males have a significant increase of the *Z*-score activation, as compared to CT males (*p* < 0.001; Fig. 3C). The specific analysis for vulnerable and resilient showed a significant condition effect (F (5, 63) = 7.783, *p* = 0.0001), with a *Z*-score increase in PNS-vul and PNS-res males, as compared to their CT counterpart (*p* = 0.004 and *p* = 0.0194, respectively; Fig. 3D). No significant changes were instead evident in female rats exposed to stress during gestation.

A significant sex x stress interaction was also evident when investigating the dorsal hippocampus (F (1, 71) = 14.51, *p* = 0.0003). Post hoc analysis revealed that PNS males have significantly reduced *z*-scores, when compared to the CT group (*p* = 0.019; Fig. 3E). The analysis for resilient and vulnerable male rats, in line with the overall PNS effect, shows a significant modulation (F (5, 66) = 8.813, *p* < 0.0001), with a significant reduction of the *Z*-score in PNS-vul and PNS-res males, as compared to CT (*p* = 0.0147 and *p* = 0.0067, respectively; Fig. 3F). Even tough female animals show an overall increase on z-activation of this brain region, such effect did not reach statistical significance when considering the whole PNS group, as well as the PNS-vul and PNS-res subgroups.

Last, the analysis of the ventral hippocampus shows a significant effect of PNS (F (1, 74) = 4.021, *p* = 0.0486) as well as of sex (F (1, 74) = 74.97, *p* < 0.0001) (Fig. 3G). We found that female animals have a significantly lower *Z*-score activation compared to males, and that a reduction was also evident in PNS rats compared to their CT counterpart. When considering the cluster analysis, we found a significant effect of condition (F (5, 69) = 3.621, *p* = 0.0057; Fig. 3H). Post hoc analysis shows that PNS-res male animals have decreased activation score when compared to both CT (*p* = 0.002) and PNS-vul (*p* = 0.0133) rats. Even tough females appear to have a similar modulation in this brain region, such changes did not reach statistical significance.

**Fig. 4:**
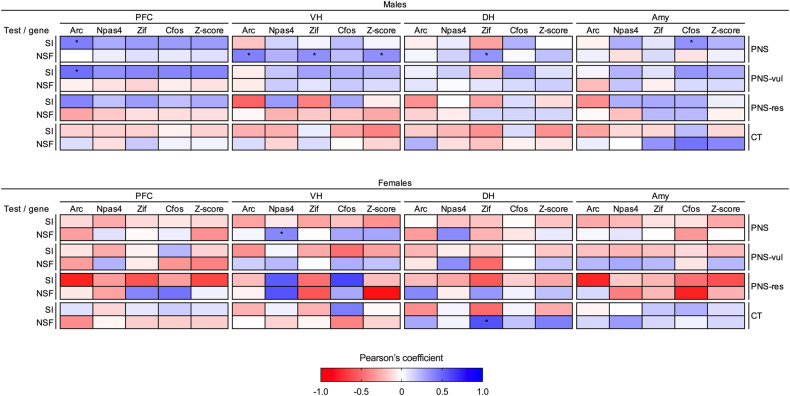
Summary of the Pearson coefficient values between the analyzed phenotypical traits and gene expression levels. Matrix displays the correlations between the behavioral phenotype (social preference from the social interaction test and latency to eat from the novelty suppressed feeding test) and IEGs expression in the prefrontal cortex (PFC), ventral hippocampus (VH), dorsal hippocampus (DH), and amygdala (Amy) in male (top panel) and female (bottom panel) rats, considering the overall effect of prenatal stress exposure (PNS) or the vulnerable (PNS-vul) and resilient (PNS-res) subgroups. Genes are shown individually (Arc, Npas4, Zif-268, Cfos) and as a global activation score (*Z*-score). Blue indicates positive correlations while red indicates negative correlations. Significant correlations are reported as *p < 0.05.

To establish whether the transcriptional changes of activity-dependent genes observed across the four brain areas could represent an indicator of a specific behavioral dysfunction, we investigated the correlation between the behavioral phenotype and each IEG mRNA levels in both male and female animals using the Pearson’s coefficient (Fig. 4). While there was no evident correlation between behaviors and gene expression data in the brain of control male animals, the changes of IEGs expression in the ventral hippocampus of PNS male animals was positively correlated with the performance in the NSF test (r = 0.44; *p* = 0.02), suggesting that higher expression levels correlated with higher latency time during the task. Although not statistically significant, we could observe a similar positive correlation between the gene expression levels in the ventral hippocampus of PNS-vul animals and the behavioral outcome in the NSF test. On the contrary, a negative not significant correlation was found between the expression of activity-dependent genes and the anxiety-related behavior of PNS-res and CT rats. Social behaviors of male animals did not correlate with IEG mRNA levels in any brain region. However, although speculative, we observed an overall opposite direction between PNS-vul and CT males, while PNS-res shows a pattern more similar to the CT group. Regarding female animals, no tissue-specific and behavior-specific patterns were observed in our correlation analyses.

Next, in order to obtain a more global picture of the effects produced by PNS exposure on brain function, we performed an integration analysis using the *Z*-score of the IEGS to investigate potential changes in the interconnectivity between different brain regions. When comparing CT animals with PNS-res and PNS-vul we could observe a similar overall reduction in the degree of co-activation between brain areas, as shown by the red lines (Fig. S5). This indicates that the relationship of activation of PNS animals (PNS-res and PNS-vul) is lower when compared to the CT group. However, PNS-vul shows a positive relationship of co-activation between DH and VH (Z_obs_ = 0.57) as compared to CT animals, which is opposite in PNS-res animals (Z_obs_ = −0.34). Indeed, when comparing the co-activation between brain areas in PNS-vul and PNS-res, it is possible to observe a global positive relationship, suggesting that PNS-vul may show a stronger interconnectivity between brain regions compared to PNS-res, an effect that is primarily driven by the interaction between VH-DH (Z_obs_ = 0.8) and DH-AMY (Z_obs_ = 0.89) connections.

### Analysis of excitation and inhibition markers in the ventral hippocampus of PNS vulnerable and resilient animals

Based on the previous results, we next focused on the ventral hippocampus where we found a significantly different activation pattern between animals that were vulnerable or resilient to PNS exposure. Since the altered activation of a given brain region may originate from an altered excitatory/inhibitory balance, we decided to investigate the mRNA levels of Vglut1 (as an excitatory marker) and Vgat (as an inhibitory marker).

**Fig. 5:**
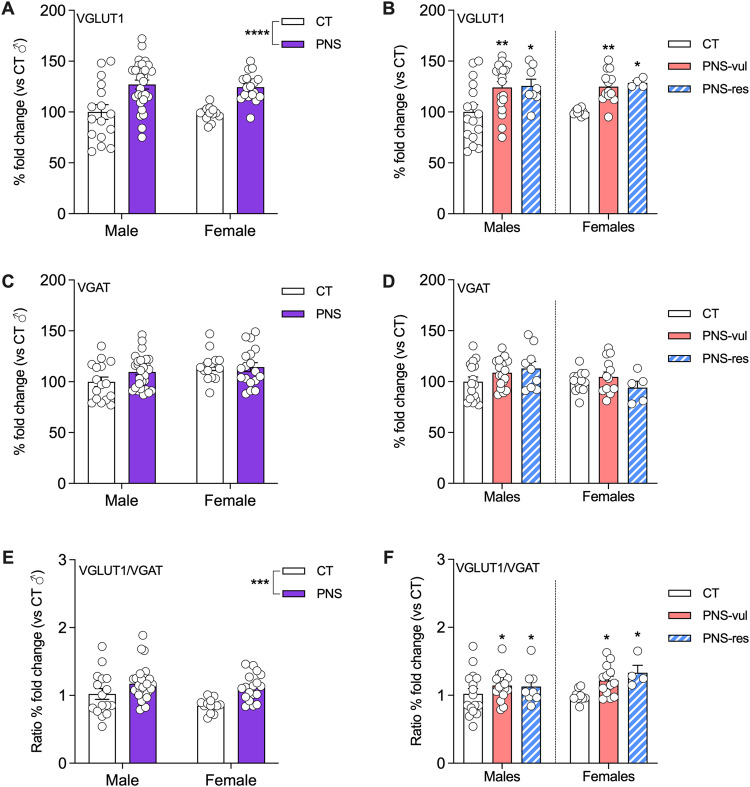
Effect of PNS exposure on the expression of excitatory and inhibitory markers in the ventral hippocampus in male and female adolescent offspring. The analyses of VGLUT1 and VGAT were performed as whole PNS group (panels **A**, **C** and **E**) or after the separation in vulnerable (PNS-vul) and resilient (PNS-res) to the gestational manipulation (**B**, **D** and **F**). The data shows mean ± SEM of *n* = 14 to 30 per group for panels **A**, **C** and **E** whereas of *n* = 5 to 19 per group for panels **B**, **D** and **F**. Statistical analysis (panels **A**, **C**, and **E**): two-way ANOVA, PNS effect, ****p < 0.0001, ***p < 0.001. Statistical analysis (panels **B**, **D**, and **F**): one-way ANOVA, Tukey’s post hoc, *p < 0.05, **p < 0.01, statistically different from the respective CT group.

With respect to Vglut1, we found a significant stress effect (F (1, 71) = 27.64, *p* < 0.0001; Fig. 5A), with PNS animals, both male and female, expressing higher mRNA levels of this gene. When considering vulnerable and resilient animals, there was a significant effect of the condition (F (5, 63) = 4.617, *p* = 0.0012; Fig. 5B). Indeed, both male and female PNS-vul and PNS-res show increased Vglut1 expression, when compared to the respective CT group (*p* < 0.01 and *p* < 0.05 for both sexes, respectively). Conversely, the analysis of Vgat mRNA levels did not show any significant differences among the experimental groups (Figs. 5C, D). We also have calculated the ratio between Vglut1 and Vgat expression as an indication of the excitatory/inhibitory (E/I) balance within this brain area. As expected by the analysis of the single genes, we found a significant stress effect (F (1, 71) = 12.99, *p* = 0.0006; Fig. 5E), with PNS animals, independently of sex, showing a higher ratio, when compared to CT animals. Moreover, the significant effect was also evident in the analysis of vulnerable and resilient animals (F (5, 62) = 2.205, *p* = 0.042; Fig. 5F), with both PNS-vul and PNS-res showing a significantly higher excitatory/inhibitory ratio when compared to CT animals (*p* < 0.05 for all comparisons).

### Analysis of glucocorticoid-related markers in the ventral hippocampus of PNS vulnerable and resilient animals

Next, considering the link between the long-term effects of early life stress and the glucocorticoid system, we decided to investigate if the reduced activity state in resilient rats was associated with altered expression of stress related genes. To this purpose we analyzed the expression of the glucocorticoid receptor Nr3c1 (also known as GR) as well as of one of its downstream transcriptional targets, namely Serum/Glucocorticoid Regulated Kinase 1 (Sgk1).

**Fig. 6:**
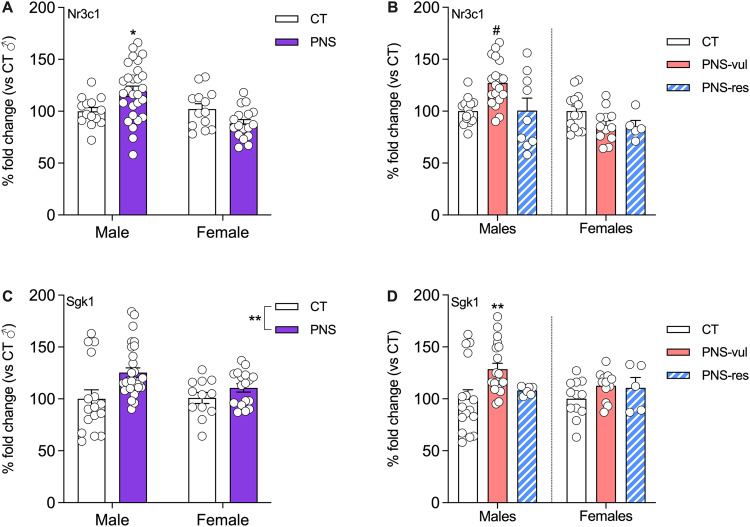
Effect of PNS exposure on the expression of glucocorticoid-related markers in the ventral hippocampus in male and female adolescent offspring. The analysis of Nr3c1 (glucocorticoid receptor) and Sgk1 were performed as whole PNS group (panels **A**, **C**) or after the separation in vulnerable (PNS-vul) and resilient (PNS-res) to the gestational manipulation (panels **B**, **D**). The data shows mean ± SEM of *n* = 14 to 30 per group for panels (**A**) and (**C**) whereas of *n* = 5 to 19 per group for panels (**B**) and (**D**). Statistical analysis: two-way ANOVA, PNS effect, **p < 0.01; Tukey’s post hoc, *p < 0.05, statistically different from CT male. Statistical analysis (panels **B**, **D**): one-way ANOVA, Tukey’s post hoc, **p < 0.01, statistically different from CT male, ^#^*p* < 0.05, statistically different from CT and PNS-res male groups.

With respect to the expression of Nr3c1, we found a significant stress x sex interaction (F (1, 68) = 10.32, *p* = 0.002; Fig. 6A). *Post hoc* analysis revealed that PNS male rats show a significant up-regulation of its mRNA levels when compared to controls (*p* = 0.0318). Moreover, a significant condition effect was found when considering vulnerable and resilient animals (F (5, 68) = 5.281, *p* = 0.0004; Fig. 6B), although only PNS-vul males show increased expression of Nr3c1 when compared to controls (*p* = 0.031) as well as to PNS-res rats (*p* = 0.0492). Conversely, female animals did not show any significant modulation of the mRNA levels for Nr3c1.

Regarding Sgk1, we found a significant effect of stress (F (1, 71) = 8.381, *p* = 0.005; Fig. 6C), with PNS rats, independently of sex, showing higher mRNA levels of this gene. The analysis considering vulnerable and resilient animals also showed a significant condition effect (F (5, 67) = 3.615, *p* = 0.0059; Fig. 6D). However, only PNS-vul male animals showed a significant up-regulation of Sgk1 expression, as compared to the CT male group (*p* = 0.0036). Conversely, no significant changes of Sgk1 expression were found in female animals.

## Discussion

Exposure to stress early in life can lead to long lasting behavioral and neurobiological consequences, which may enhance the susceptibility for mental disorders [25–27]. In the present study, we found that exposure to PNS leads to emotional dysregulation in adolescence, an important temporal window for the onset of psychiatric disorders [19], although such impairment can be observed only in a percentage of animals (vulnerable), whereas a subgroup of them appears to be resilient. To the best of our knowledge, this is the first demonstration of a stratification of the behavioral impairment based on different psychopathological domains after stress exposure during gestation using a statistical approach. Furthermore, we identified molecular changes that are specifically associated with resilience to gestational stress. In detail, resilient male animals show reduced activity of the ventral hippocampus, a finding that is supported by an integrated analysis that showed a correlation between the hippocampal activity state and the behavioral performance. However, we also identified changes in both vulnerable and resilient animals, such as the altered activation of the amygdala and the dorsal hippocampus. This may be particularly important since it points to an altered functional state regardless of the behavioral phenotype, suggesting that animals that do not show a behavioral phenotype (resilient) are distinct from vulnerable animals, but they may also differ from control animals, thus representing an intermediate phenotype that, speculatively, could respond differently to subsequent adversities.

Animals exposed to PNS showed significant behavioral differences that resemble specific psychopathologic domains, with reduced sociability, increased anxiety and anhedonic behavior. These data are in line with previous reports showing the negative effects of different paradigms of prenatal stress on behavior outcomes [28–31]. It is interesting that the cluster analysis used to identify vulnerable and resilient animals revealed that the tests related to anxiety and sociability had higher predictive importance than the depressive one. This may suggest that during adolescence, anxiety and social impairment may reflect an early disability associated with the exposure to traumatic experiences during gestation. Anxiety disorders, including social phobia and others, usually have its first onset during childhood and early adolescence [17]. When analyzing those rats separately for social and anxious behaviors we found that only vulnerable rats, both male and female, show the affected phenotype. Conversely, the cluster analysis applied to the anhedonic-like behavior was not able to identify subgroups of animals with a different response to PNS exposure. Interestingly, not all animals classified as vulnerable or resilient show the same pattern of alterations in the three behavioral tests (data not shown).

At the molecular level, we demonstrate that male animals resilient to PNS exposure show decreased activity of the ventral hippocampus (VH), as compared to CT animals, whereas vulnerable rats did not show any significant difference. Interestingly, recent studies have shown that adult mice resilient to chronic social defeat or to chronic variable stress show reduced VH activity [32, 33] and that VH inhibition can improve the social deficits and anxiety-like behavioral phenotype after the chronic stress exposure [34]. These data suggest that reduced activity of this region may contribute to resilience, whereas a stress-induced increase of the neuronal activity can be observed in vulnerable animals. Although there are methodological differences when compared to the present study, it may be inferred that a lower activity state found in a subgroup of adolescent rats may be relevant for their behavioral resilience to PNS exposure. Furthermore, while we did not observe significant behavioral difference between males and females, it is important to note that the reduction of VH activity in female rats resilient to PNS did not reach statistical significance. This may be due to the low number of PNS-res females or, alternatively, it may suggest a sexual dimorphism in the molecular mechanisms associated with the resilience to PNS exposure.

The different activity state in the VH of resilient males does not appear to be a consequence of an excitatory/inhibitory imbalance. We found that PNS male and female rats, both vulnerable and resilient, have increased VGlut1 expression in the VH, leading to an elevated E/I ratio, which may represent a negative consequence of PNS exposure. Indeed, hyperexcitability due to a disrupted E/I balance has been associated with behavioral alterations after stress exposure [35]. Moreover, early life adversity can shape the function of different neural circuits [23]. On these bases, the excitability of VH can be modulate by afferents from the entorhinal cortex [36], also with the contribution of adult-born granule cells that are known to confer stress resilience by inhibiting the ventral dentate gyrus [34]. Interestingly, a reduction of neurogenesis has been shown in adult rats previously exposed to PNS, although it is not known if this may occur in adolescent animals [37]. These mechanisms can also be modulated by serotonin inputs through different serotonin receptors in the dentate gyrus, including the 5HT1A subtype [38].

Our recent meta-analysis has demonstrated that prenatal stress leads to long-lasting effects on the HPA axis function [39]. Indeed, glucocorticoid-related mechanisms may also contribute to the long-term changes produced by early in life stress exposure [23]. Our data shows that PNS-vul male rats display a significant upregulation in the expression of glucocorticoid receptors (GR) and of one of its target genes, Sgk1. An overexpression of GR in transgenic models has been shown to induce anxiety and depressive-like behavior in rodents [40] suggesting that, at least in male animals, such changes may contribute to the behavioral abnormalities found in rats vulnerable to PNS exposure.

We also show that there are significant brain changes due to the exposure to gestational stress, independently from the presence of emotional dysregulation (vulnerability). Indeed, we found that both PNS resilient and vulnerable males show decreased activity of the dorsal hippocampus. The lack of phenotype specificity may be due to the fact that our classification was based on emotional-related behaviors, and the DH is a key brain region for cognitive processes [41]. Interestingly, we have previously shown that adult rats exposed to gestational stress show reduced working memory in the object recognition test [13]. Furthermore, male PNS rats, independently from vulnerability or resilience, show increased activity state of the amygdala, an effect that has been previously associated with stress exposure [42, 43]. Indeed, exposure to chronic stress results in a hyperactivation of the amygdala, possibly linked to an increased excitability of pyramidal neurons in this same brain region [44].

The effects of PNS exposure observed in adolescent rats may be due to reduced maternal care, which is known to produce biological and behavioral impairments later in life [45–47]. Indeed, PNS dams showed decreased frequency of arched-back nursing, the most important and efficient position for the pups’ proper lactation [47], together with increased exits from the nest that leads to a fragmentation of the maternal care compared to CT dams. Accordingly, other protocols of early life stress, as the limited bedding and nesting, also show a disruption of maternal care [46, 48, 49]. Although no analysis was performed in the dams during the PNS period, it is already known that this manipulation can increase the levels of glucocorticoid receptors and decrease the levels of the enzyme 11β-hydroxysteroid dehydrogenase (11β-HSD2) in the placenta, which might affect the maternal behavior and contribute to the behavioral phenotype of the offspring [50].

While our study has tried to address key questions related to the effects of PNS with a detailed examination of emotional-related behavior in male and female offspring and correlating the molecular changes with the behavioral outcomes, there are limitations that must be acknowledged. First, animals were not evaluated for possible cognitive impairments, which may also represent an important disease domain for stress-associated disorders. Next, we did not check the estrous cycle of female animals, which may affect behavioral performances. Also, the animals were only tested in adolescence, and it will be important to establish if and how the phenotype observed at this age could evolve after the transition to adulthood. Moreover, while there were no differences in PNS-induced emotional dysregulation between males and females, our target molecular analyses failed to identify significant molecular alterations in the female subgroups. Additionally, we did not assess whether the alterations in mRNA expression are associated with changes at protein level. Last, it will be important to establish if and how PNS-vul and PNS-res animals will show a different responsiveness to subsequent environmental challenges.

Overall, we showed that stress exposure during gestation produces emotional dysregulation only in a sub-group of adolescent male and female offspring. The characterization of the neurobiological mechanisms contributing to resilience or vulnerability to stress will be instrumental to identify clinical biomarkers as well as mechanisms that may be targeted by therapeutic approaches aimed at counteracting specific pathologic domains of mental disorders, including major depression and schizophrenia.

### Supplementary information

Supplementary material

## Data Availability

Data will be made available upon request.
