# Supplementary material for: Vulnerability and resilience to prenatal stress exposure: behavioral and molecular characterization in adolescent rats
Source: Transl Psychiatry. 2023 Nov 22;13:358. doi: 10.1038/s41398-023-02653-6 (PMC10665384; doi:10.1038/s41398-023-02653-6)
Supplement: Supplementary file 1 — Supplementary material [file 41398_2023_2653_MOESM1_ESM.docx]

SUPPLEMENTARY MATERIAL

**Vulnerability and resilience to prenatal stress exposure: behavioral and molecular characterization in adolescent rats.**

Kerstin Camile Creutzberg^1^*, Veronica Begni^1^*, Rodrigo Orso^1^, Francisco Sindermann Lumertz^2^, Luis Eduardo Wearick-Silva^2^, Saulo Gantes Tractenberg^2^, Moira Marizzoni^3,4^, Annamaria Cattaneo^1,3^, Rodrigo Grassi-Oliveira^2,5^, Marco Andrea Riva^1,3^

*first co-authorship

^1^Department of Pharmacological and Biomolecular Sciences, University of Milan, Milan, Italy.

^2^School of Medicine, Pontifical Catholic University of Rio Grande do Sul, Porto Alegre, RS, Brazil

^3^Biological Psychiatry Unit, IRCCS Istituto Centro San Giovanni di Dio Fatebenefratelli, Brescia, Italy.

^4^Lab of Neuroimaging and Alzheimer’s Epidemiology, IRCCS Istituto Centro San Giovanni di Dio Fatebenefratelli, Via Pilastroni, 4, Brescia 25125, Italy

^5^Translational Neuropsychiatry Unit, Department of Clinical Medicine, Aarhus University, Aarhus, Denmark

**METHODS**

**Analysis of maternal behavior**

Maternal behavior was assessed in intercalated days between PND1 and PND9. Observations were conducted in three blocks of 20 min each, starting at 9 am, 1 pm, and 5 pm ± 30min. One observation was made every 2 min with a total of 33 observations per day. The frequency of the following behaviors was evaluated: arched-back nursing, blanket or passive nursing, licking and grooming, off-nest, self-grooming, eating, drinking, and the number of exits from the nest. The first three abovementioned behaviors were classified as pups-directed behaviors while the remaining, except for the exits from the nest, were classified as self-directed behaviors. Results are presented as the average of all days.

**Social interaction test**

At PND35 animals were exposed to the social interaction test. The test was divided into two phases, habituation and test. During the habituation phase, the animal was placed in the plexiglas arena (1m x 1m) which contained an empty grided enclosure and had 3 minutes to freely explore it. After the 3 minutes, the animal was removed and moved to a residential cage while an unfamiliar animal (matching sex, age, and weight) was placed inside the grided enclosure. Right after, the tested animal was replaced in the arena for the test phase which also lasted 3 minutes. During this time the animal was again free to explore the apparatus and the unfamiliar animal. The grided enclosure allowed nose and sensorial contact between animals. The total time of interaction (sniffing, exploring, or touching) between animals was evaluated. Results are shown as percentage calculated as follows: time spent interacting with the unfamiliar animal divided by the sum of the time spent with the unfamiliar animal and the empty grided enclosure.

**Sucrose preference test**

Immediately after the end of the SI test, animals were placed in a new residential cage with two bottles of water for habituation. The night before the test (PND37), animals were water deprived for 12h. On the day of the test (PND38), both bottles were replaced, one containing the usual drinking water and the other containing 1% sucrose solution. Animals were free to choose from which bottle to drink for a period of 3 hours. The position of the bottles was swapped halfway through the test to avoid a side preference. Bottles were weighed before and after the test and the sucrose preference was calculated based on the intake (in grams) of both water and sucrose. Preference was calculated as follows: total sucrose intake divided by the sum of total sucrose and water intakes.

**Novelty suppressed feeding test**

Lastly, on PND 39, the novelty-suppressed feeding test was performed. Following 12 hours of food deprivation, rats were placed in the corner of the testing arena (the same one used for the SI test), which contained a single food pellet right in the center. The time to approach and eat the pellet was measured during a 5min period, however, if the animal ate the food the timer was stopped, and the animal was removed and placed back in the residence cage.

**Table S1. Primers and probes ID’s or sequences**

| Gene | Sequence primer forward | Sequence primer reverse | Sequence probe |
| --- | --- | --- | --- |
| ß-Actin | CACTTTCTACAATGAGCTGCG | CTGGATGGCTACGTACATGG | TCTGGGTCATCTTTTCACGGTTGGC |
| VGLUT1 | ACTGCCTCACCTTGTCATG | GTAGCTTCCATCCCGAAACC | CTTTCGCACATTGGTCGTGGACATT |
| VGAT | ACGACAAACCCAAGATCACG | GTAGACCCAGCACGAACATG | TTCCAGCCCGCTTCCCACG |
|  | | | |
| Gene | ID | | |
| GAPDH | Rn99999916_s1 | | |
| Arc | Rn00571208_g1 | | |
| Npas4 | Rn01454622_g1 | | |
| Zif-268 | Rn00561138_m1 | | |
| Cfos | Rn02396760_g1 | | |
| Nr3c1 | Rn00561369_m1 | | |
| Sgk1 | Rn00570285_m1 | | |

**RESULTS**

**Analysis of activity-regulated genes in the prefrontal cortex of PNS vulnerable and resilient animals**

Regarding the analysis of the single IEGs in the prefrontal cortex (PFC), limited modulations due to PNS exposure were found. When considering Arc, a significant sex x PNS interaction was found (F (1, 61) = 11.07, *p* = 0.0015; Fig. S1A). *Post hoc* analysis showed that female PNS animals show higher expression of this gene compared to female CT animals. The analysis of vulnerable and resilient showed a significant condition effect (F (5, 57) = 7.331, *p* < 0.0001; Fig. S1B), as female PNS-vul and PNS-res have increased expression of Arc, as compared to CT (*p* = 0.02 and *p* = 0.0033, respectively). Next, a sex effect was found for Npas4 expression (F (1, 63) = 19.00, *p* < 0.0001; Fig. S1C), with decreased mRNA levels in females, as compared to males, although the cluster analysis with PNS-vul and PNS-res did not show any significant changes (Fig. S1D). With regard to Zif-268 and c-Fos, exposure to PNS did not produce any significant difference in males and females (Fig. S1E and G), even when considering the PNS-vul and PNS-res subgroups (Fig. S1F and H).

**Analysis of activity-regulated genes in the amygdala of PNS vulnerable and resilient animals**

Considering the amygdala, the analysis of Arc expression revealed a significant sex x PNS interaction (F (1, 66) = 4.580, *p* = 0.036; Fig. S2A). *Post hoc* analysis showed that PNS male animals have increased expression of this gene, as compared to CT males (*p* = 0.0038) and PNS females (*p* = 0.0105). The analysis of vulnerable and resilient subgroups showed a significant condition effect (F (5, 62) = 5.385, *p* = 0.0004; Fig. S2B), since PNS-vul males have increased Arc mRNA levels, as compared to CT males (*p* = 0.0043). Regarding Npas4, an sex x PNS interaction was found (F (1, 64) = 4.672, *p* = 0.0344; Fig. S2C). *Post hoc* analysis revealed increased Npas4 expression in PNS males, as compared to the CT counterpart (*p* = 0.0017) and to PNS females (*p* = 0.0249). The analysis for vulnerable and resilient animals showed a condition effect (F (5, 61) = 5.429, *p* = 0.0003; Fig. S2D), where PNS-vul (*p* = 0.0287) and PNS-res (*p* = 0.0061) display increased Npas4 mRNA levels vs. CT males. Next, Zif-268 analysis showed a sex x PNS interaction (F (1, 68) = 7.938, *p* = 0.0063; Fig. S2E). *Post hoc* analysis revealed increased Zif-268 expression in PNS males, as compared to CT (*p* = 0.0104). The analysis for vulnerable and resilient animals showed a condition effect (F (5, 62) = 4.218, *p* = 0.0023; Fig. S2F), with PNS-vul (*p* = 0.0131) having higher Zif-268 expression than CT. Lastly, for c-Fos analysis only a sex effect was found (F (1, 60) = 11.41, *p* = 0.0013; Fig. S2G), with increased expression in females. For the sub-cluster analysis, a condition effect was observed (F (5, 53) = 5.452, *p* = 0.0004; Fig. S2H), with both PNS-vul (*p* = 0.0303) and PNS-res (*p* = 0.0031) showing increased mRNA levels of c-Fos than CT male animals.

**Analysis of activity-regulated genes in the dorsal hippocampus of PNS vulnerable and resilient animals**

Regarding dorsal hippocampus, the analysis of Arc expression revealed a sex x PNS interaction (F (1, 66) = 9.846, *p* = 0.0025; Fig. S3A). *Post hoc* analysis showed that PNS males have lower mRNA levels than CT (*p* = 0.008). The analysis of vulnerable and resilient animals shows a significant condition effect (F (5, 62) = 5.968, *p* = 0.0001; Fig. S3B), with PNS-res males having decreased expression as compared to CT animals (*p* = 0.0211). Also, Npas4 show a sex x PNS interaction (F (1, 68) = 8.947, *p* = 0.0039; Fig. S3C). Post hoc analysis revealed that PNS male animals have decreased Npas4 expression as compared to CT (*p* = 0.0157). When considering vulnerable and resilient animals, a condition effect was observed (F (5, 66) = 8.019, *p* < 0.0001; Fig. S3D), with PNS-vul females showing higher Npas4 mRNA levels than CT (*p* = 0.0223). The overall analysis of Zif-268 reported no significant PNS or sex modulation (Fig. S3E). However, when looking at vulnerable and resilient animals, a significant condition effect was observed (F (5, 65) = 3.404, *p* = 0.0086; Fig. S3F), with PNS-res males having reduced Zif-268 expression than CT animals (*p* = 0.0158). Lastly, c-Fos analysis show a significant sex effect (F (1, 69) = 12.93, *p* = 0.0006; Fig. S3G), with decreased expression in females, whereas no significant alterations were found when considering the sub-cluster of vulnerable and resilient (Fig. S3H).

**Analysis of activity-regulated genes in the ventral hippocampus of PNS vulnerable and resilient animals**

Considering the ventral hippocampus, we found a significant sex effect for Arc expression (F (1, 73) = 29.00, *p* < 0.0001; Fig. S4A), with females showing lower mRNA levels than males. For the cluster analysis, a significant condition effect was seen (F (5, 67) = 2.868, *p* = 0.0209; Fig. S4B). In detail, PNS-res male animals have reduced Arc expression, as compared to CT males (*p* = 0.0049) and to PNS-vul males (*p* = 0.038). Next, the analysis of Npas4 revealed a sex (F (1, 74) = 24.88, *p* < 0.0001) and a PNS effect (F (1, 74) = 7.202, *p* = 0.009; Fig. S4C), with reduced expression in females as well as in PNS-exposed animals. When looking at stress vulnerable and resilient animals no significant changes were found (Fig. S4D). With regard to Zif-268, we observed a significant effect of sex (F (1, 72) = 27.11, *p* < 0.0001; Fig. S4E), since female animals show lower expression of this gene, as compared to males. When considering the sub-clusters of vulnerable and resilient animals, no significant changes were seen (Fig. S4F). Last, the analysis of c-Fos mRNA levels showed a significant effect of sex (F (1, 69) = 33.85, *p* < 0.0001) and of PNS (F (1, 69) = 8.156, *p* = 0.0057; Fig. S4G). In detail, both females and PNS-exposed animals have lower expression of this gene. On the other end, the analysis of vulnerable and resilient animals did not show any significant alterations (Fig. S4H).


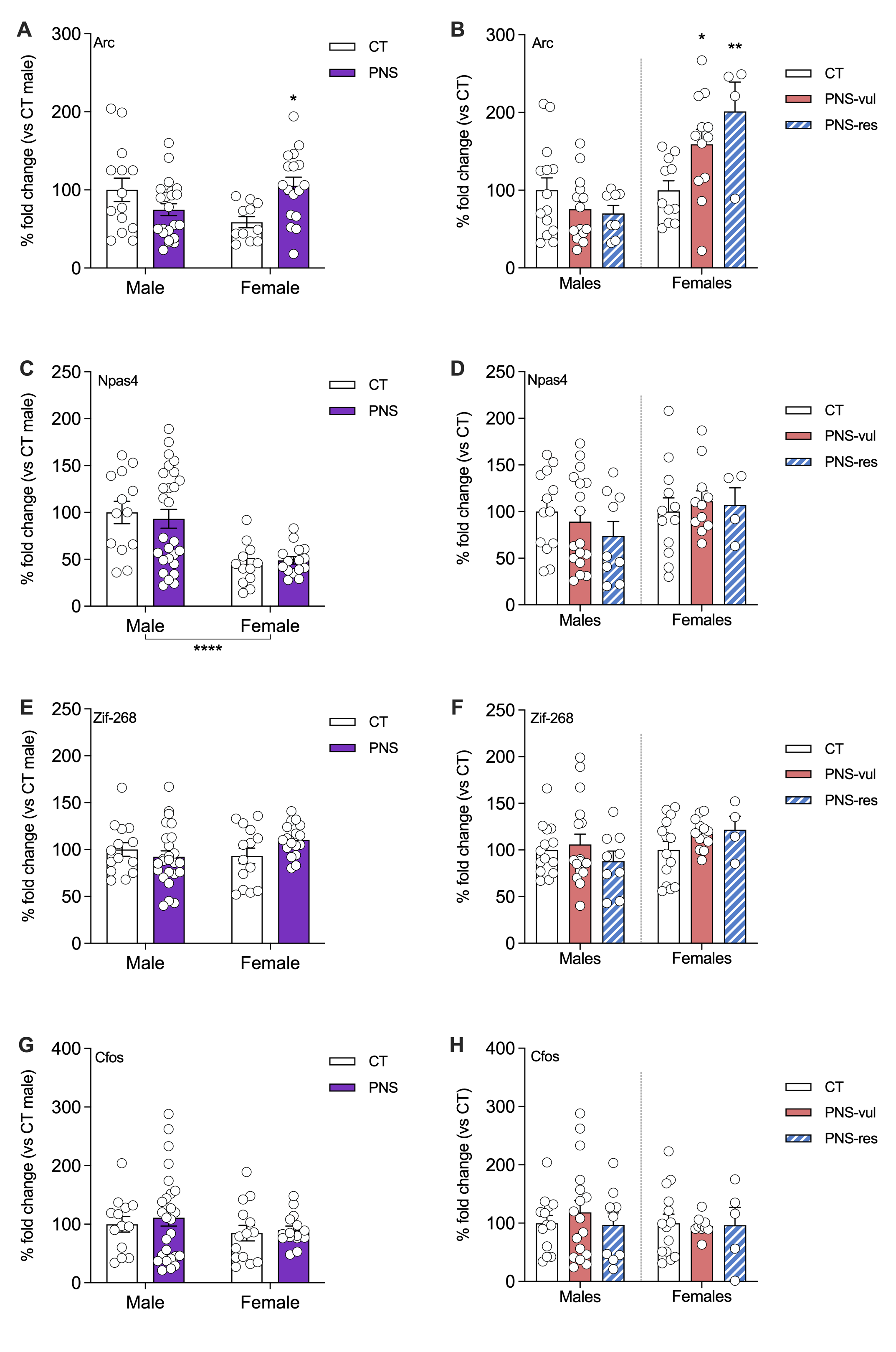


**Figure S1. Analysis of the mRNA levels of activity-regulated genes in the prefrontal cortex of adolescent offspring following PNS exposure**

The data shows the mean ± SEM for the mRNA levels of Arc (panels A and B), Npas4 (panels C and D), Zif (panels E and F), and Cfos (panels G and H). The analyses were performed as whole PNS group (A, C, E, and G) or after the separation in vulnerable (PNS-vul) and resilient (PNS-res) to the gestational manipulation (B, D, F, and H). Statistical analysis for panels A, C, E, and G): two-way ANOVA, sex effect, ****p < 0.0001; Tukey’s post hoc, *p < 0.05, statistically different from CT female (n = 14 to 30 per group). Statistical analysis for panels B, D, F, and H): one-way ANOVA, Tukey’s post hoc, *p < 0.05, **p < 0.01, statistically different from CT female (n = 5 to 19 per group).


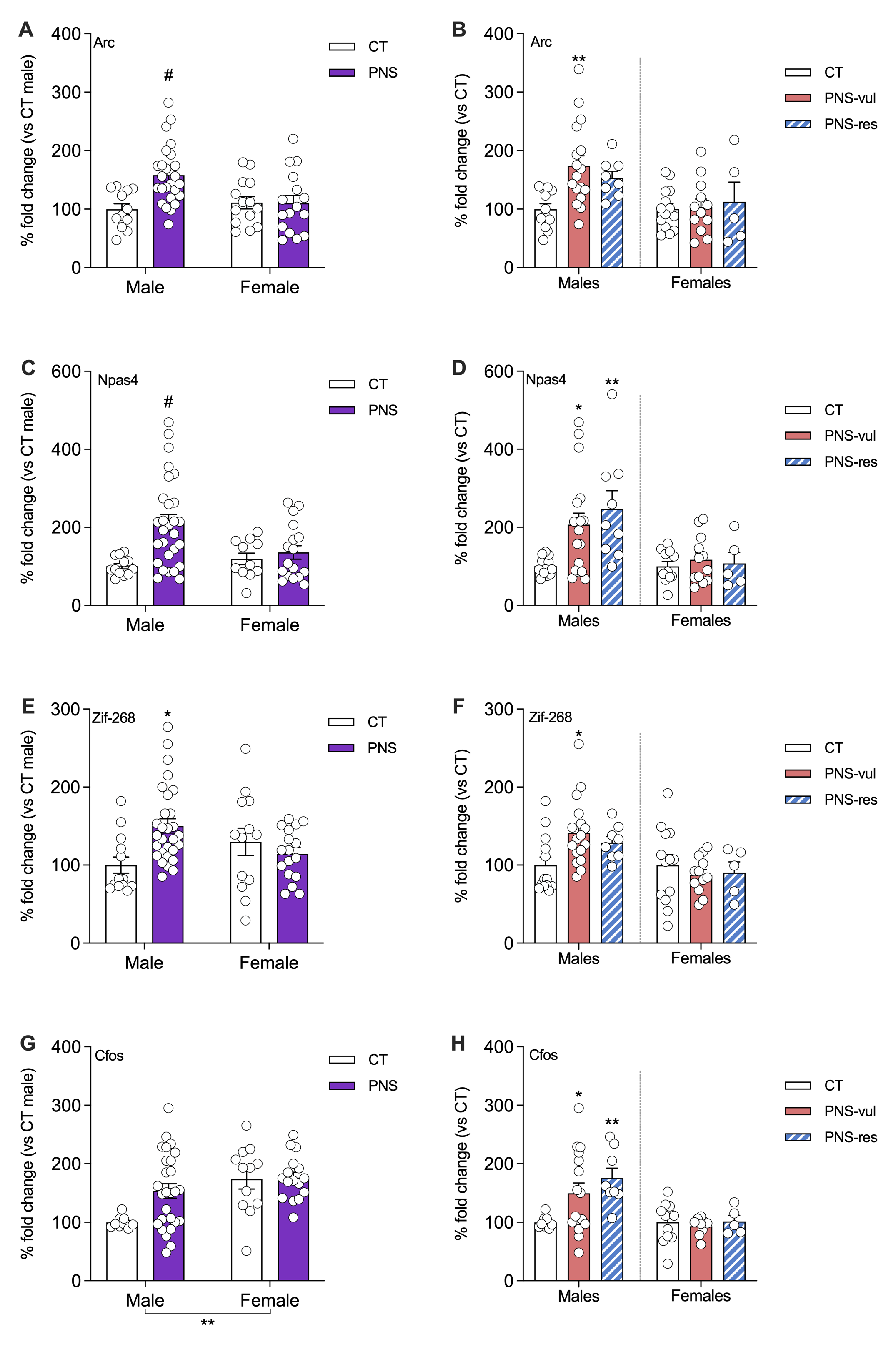


**Figure S2. Analysis of the mRNA levels of the activity-regulated genes in the amygdala of adolescent offspring following PNS exposure**

The data shows the mean ± SEM for the mRNA levels of Arc (panels A and B), Npas4 (panels C and D), Zif (panels E and F), and Cfos (panels G and H). The analyses were performed as whole PNS group (A, C, E, and G) or after the separation in vulnerable (PNS-vul) and resilient (PNS-res) to the gestational manipulation (B, D, F, and H). Statistical analysis for panels A, C, E, and G: two-way ANOVA, sex effect ***p* < 0.01; Tukey’s *post hoc*, **p* < 0.05, statistically different from CT male, ^#^p < 0.05, statistically different from every other group (*n* = 14 to 30 per group). Statistical analysis for panels B, D, F, and H: one-way ANOVA, Tukey’s *post hoc,* **p* < 0.05, ***p* < 0.01, statistically different from CT male (*n* = 5 to 19 per group).


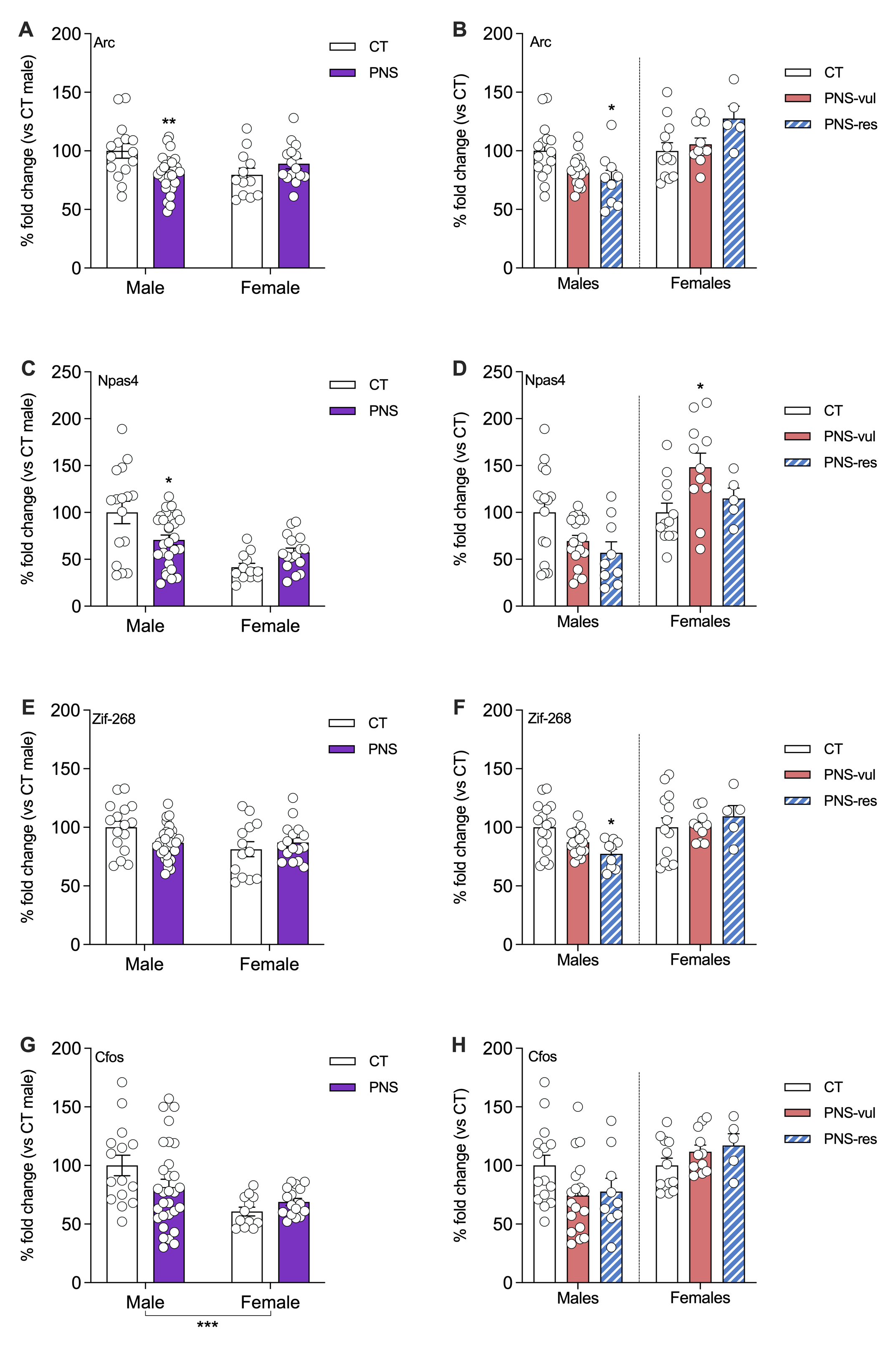


**Figure S3. Analysis of the mRNA levels of the activity-regulated genes in the dorsal hippocampus of adolescent offspring following PNS exposure**

The data shows the mean ± SEM for the mRNA levels of Arc (panels A and B), Npas4 (panels C and D), Zif (panels E and F), and Cfos (panels G and H). The analyses were performed as whole PNS group (A, C, E, and G) or after the separation in vulnerable (PNS-vul) and resilient (PNS-res) to the gestational manipulation (B, D, F, and H). Statistical analysis for panels A, C, E, and G: two-way ANOVA, sex effect ****p* < 0.001; Tukey’s *post hoc*, **p* < 0.05, ***p* < 0.01, statistically different from CT male (*n* = 14 to 30 per group). Statistical analysis for panels B, D, F, and H: one-way ANOVA, Tukey’s *post hoc,* **p* < 0.05, statistically different from the respective CT group (*n* = 5 to 19 per group).


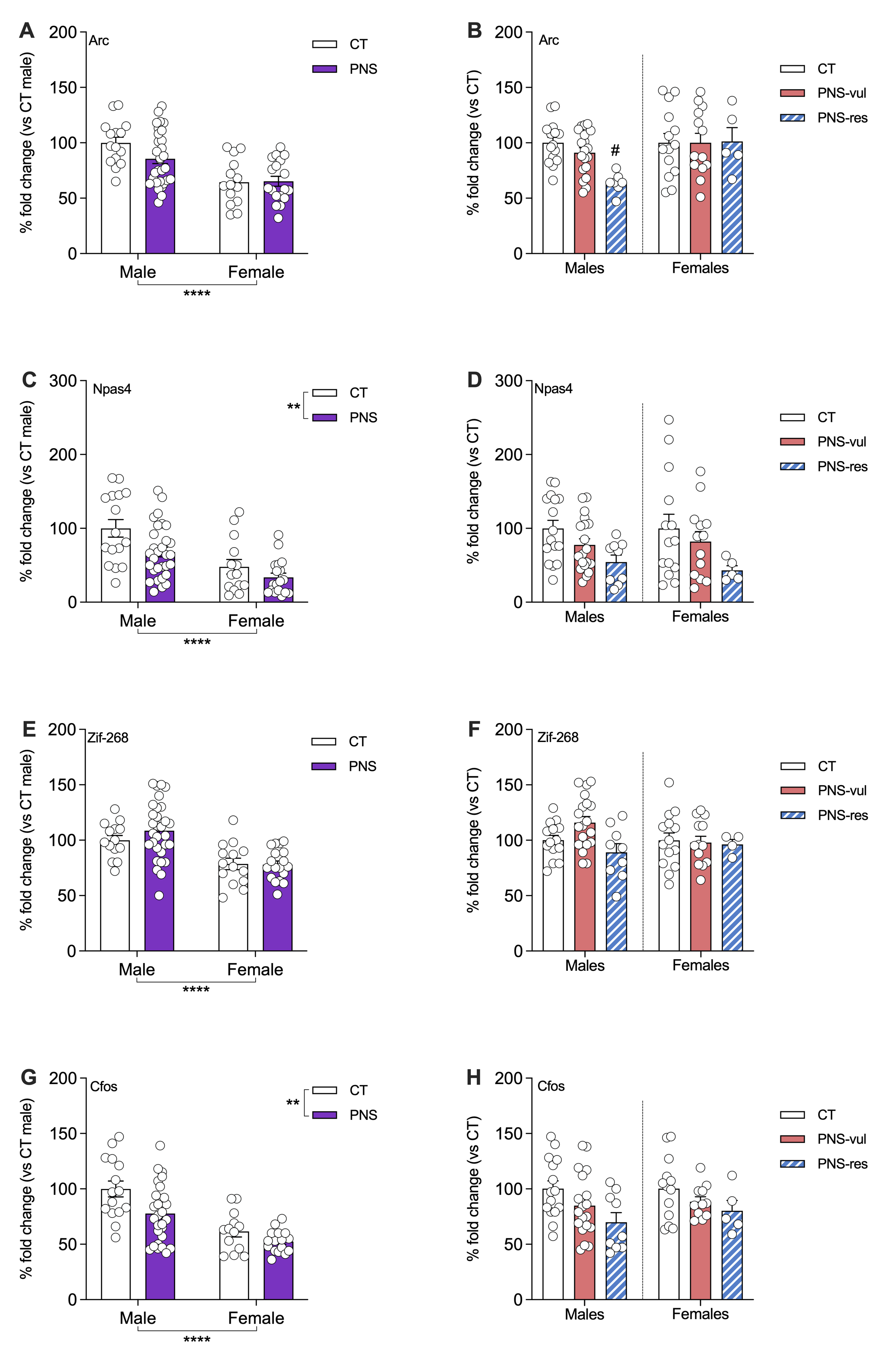


**Figure S4. Analysis of the mRNA levels of the activity-regulated genes in the ventral hippocampus of adolescent offspring following PNS exposure**

The data shows the mean ± SEM for the mRNA levels of Arc (panels A and B), Npas4 (panels C and D), Zif (panels E and F), and Cfos (panels G and H). The analyses were performed as whole PNS group (A, C, E, and G) or after the separation in vulnerable (PNS-vul) and resilient (PNS-res) to the gestational manipulation (B, D, F, and H). Statistical analysis for panels A, C, E, and G: two-way ANOVA, sex effect *****p* < 0.0001; stress effect, ***p* < 0.01 (*n* = 14 to 30 per group). Statistical analysis for panels B, D, F, and H: one-way ANOVA, Tukey’s *post hoc,* ^#^*p* < 0.05, statistically different from CT and PNS-vul male groups (*n* = 5 to 19 per group).

**
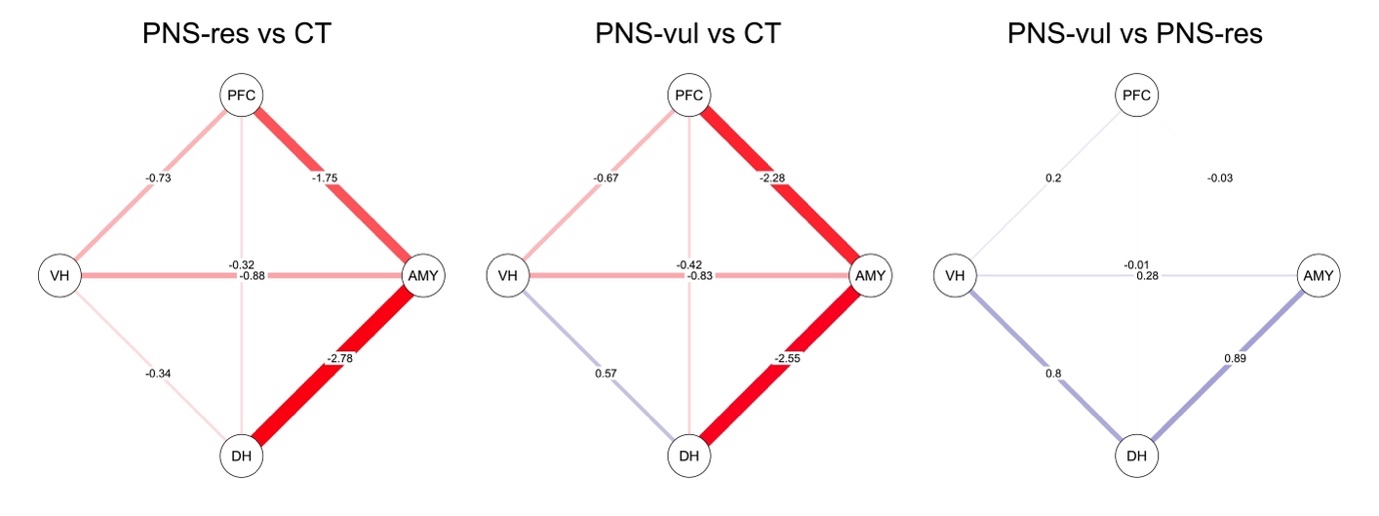
**

**Figure S5. Comparison of correlated brain activity among male animals (z-score of IEGs among all brain regions)**

The z-scores were used to calculate the z-observed (Zobs), that indicates the difference between two z-scores. The difference between two z-scores could be: Positive, indicating that the first group had a stronger or more positive relationship of co-activation between the two considered regions than the second group, illustrated by blue lines; Negative, indicating that the first group had a weaker or less positive relationship of co-activation between the two considered regions than the second group, illustrated by red lines. Line thickness represents the intensity of the difference.
